# Intragenic Suppressor of *Osiaa23* Revealed a Conserved Tryptophan Residue Crucial for Protein-Protein Interactions

**DOI:** 10.1371/journal.pone.0085358

**Published:** 2014-01-15

**Authors:** Jun Ni, Zhenxing Zhu, Gaohang Wang, Yanxia Shen, Yanyan Zhang, Ping Wu

**Affiliations:** 1 College of Life and Environmental Sciences, Hangzhou Normal University, Hangzhou, China; 2 State Key Laboratory of Plant Physiology and Biochemistry, College of Life Science, Zhejiang University, Hangzhou, China; 3 Agricultural Crops Molecular Improving Lab, Liaoning Academy of Agricultural Sciences, Shenyang, China; University of South Florida College of Medicine, United States of America

## Abstract

The Auxin/Indole-3-Acetic Acid (Aux/IAA) and Auxin Response Factor (ARF) are two important families that play key roles in auxin signal transduction. Both of the families contain a similar carboxyl-terminal domain (Domain III/IV) that facilitates interactions between these two families. In spite of the importance of protein-protein interactions among these transcription factors, the mechanisms involved in these interactions are largely unknown. In this study, we isolated six intragenic suppressors of an auxin insensitive mutant, *Osiaa23*. Among these suppressors, *Osiaa23-R5* successfully rescued all the defects of the mutant. Sequence analysis revealed that an amino acid substitution occurred in the Tryptophan (W) residue in Domain IV of Osiaa23. Yeast two-hybrid experiments showed that the mutation in Domain IV prevents the protein-protein interactions between Osiaa23 and OsARFs. Phylogenetic analysis revealed that the W residue is conserved in both OsIAAs and OsARFs. Next, we performed site-specific amino acid substitutions within Domain IV of OsARFs, and the conserved W in Domain IV was exchanged by Serine (S). The mutated OsARF(WS)s can be released from the inhibition of Osiaa23 and maintain the transcriptional activities. Expression of *OsARF(WS)s* in *Osiaa23* mutant rescued different defects of the mutant. Our results suggest a previously unknown importance of Domain IV in both families and provide an indirect way to investigate functions of OsARFs.

## Introduction

The phytohormone auxin is critical for plant growth and development, including lateral root development, embryonic development, tropic responses, apical dominance and vascular development [1]. Auxin is also involved in the crown root initiation and quiescent center maintenance in rice [2], [3], [4].

The transmission of auxin signaling is controlled by the interaction between Aux/IAA and ARF proteins [5]. Limited interaction studies suggested that, in the absence of auxin, the Aux/IAA repressors interact with ARFs and recruit co-repressors of TOPLESS (TPL) family, preventing the ARFs from regulating auxin response genes [6]. In the presence of auxin, the Aux/IAA proteins are degraded by the ubiquitin-proteasome pathway. In this process, auxin promotes the interaction between Aux/IAA proteins and TIR1 (Transport Inhibitor Response1) F-box (or its homologues) of the SCF (Skp1-Cullin-F-box) complex that acts as an auxin co-receptor [7], [8], [9], [10]. Destruction of the Aux/IAA repressors would then release the ARFs to regulate the transcription of auxin response genes [11]. Aux/IAA and ARF proteins contain a similar carboxyl-terminal domain (Domain III/IV), and Domain III/IV serve for dimerization with Aux/IAA and ARF proteins [12], [13], [14]. Most ARF proteins consist of a conserved amino-terminal DNA-binding domain that recognize TGTCTC auxin response elements in promoters of genes responding to auxin, followed by a middle region that functions as an activation domain or repression domain, and ending with Domain III/IV similar to those in the Aux/IAAs in the carboxyl-terminal domain [15].

The Aux/IAAs are short-lived nuclear proteins and generally contain four conserved domains (i.e., referred to as Domain I, II, III and IV) [16]. The rapid degradation of Aux/IAA proteins require the core sequence GWPPV at positions 4–8 in the 13-amino acid consensus sequence in Domain II [17]. The Domain II interacts with the SCF complex in an auxin-dependent manner and confers instability to the Aux/IAA proteins [14], [18]. Mutations in the core sequence of Domain II block the degradation of Aux/IAAs and interrupt the transmission of the auxin signaling pathway by constitutively suppressing ARF activity. Many auxin-insensitive mutants containing gain-of-function mutant alleles of *iaa* have been reported in rice [4], [19], [20]. Of all the *iaa* mutants, *Osiaa23* was the most interesting mutant reported in rice. *Osiaa23* exhibits pleiotropic defects in both root and shoot [4]. This implies that a number of OsARFs have been suppressed by the stabilized Osiaa23. However, the mechanisms in protein-protein interactions between Osiaa23 and OsARFs and the functions of these OsARFs are still unknown.

In this research, we isolated six intragenic suppressors of *Osiaa23*. One of these suppressors rescued all the defects of *Osiaa23*. Sequence analysis revealed that an amino acid substitution occurred in a conserved W residue in Domain IV of Osiaa23. Yeast two-hybrid experiments and analysis of transgenic plants expressing mutated *OsARF(WS)s* in the background of *Osiaa23* revealed a previously unknown importance of Domain IV in both families and provide an indirect way to investigate functions of OsARFs.

## Materials and Methods

### Plant growth conditions

Rice was grown in culture solution in growth room at temperature regimes of 28/22°C (day/night) and 70% humidity under a 12-h photoperiod. The hydroponic solution contained 3.0 mM NH_4_NO_3_, 1.0 mM CaCl_2_, 0.32 mM NaH_2_PO_4_, 0.51 mM K_2_SO_4_, 1.65 mM MgSO_4_, 3.13 mM MnCl_2_, 1.52 mM (NH_4_)_6_Mo_7_O_24_, 1.5 mM H_3_BO_3_, 1.5 mM ZnSO_4_, 1.6 mM CuSO_4_, 35 mM FeCl_3_, and 70 mM citric acid. The pH of the solution was adjusted to 5.5.

### Isolation of suppressors of *Osiaa23-3*

*Osiaa23-3* is a weak allele of *Osiaa23* in the genetic background of *indica* cultivar ‘Kasalath’. The suppressors of *Osiaa23-3* were screened from M2 population of EMS treated *Osiaa23-3* seeds. *Osiaa23-3* has no lateral roots, so 7-day-old seedlings with lateral roots were isolated as suppressors. The *OsIAA23* genes of all the suppressors were cloned and sequenced for checking the intragenic mutations. We screened 20,000 M2 plants and isolated six suppressors of *Osiaa23-3*. Sequence analysis showed that they were all intragenic suppressors.

### Yeast two-hybrid analysis

Yeast two-hybrid analysis was performed according to the instructions for the MATCHMAKER GAL4 Two-Hybrid System 3 (Clontech). The coding sequences of *Osiaa23-3* and *Osiaa23-R5* were amplified by primers *OsIAA23-U* and *OsIAA23-L*, cloned into *pGBKT7* and transformed into yeast *Y187*. The coding sequences of *OsARFs* and mutated *OsARF(WS)s* were amplified by primers *OsARF6-U* and *OsARF6-L* for *OsARF6* and *OsARF6(WS)*, *OsARF12-U*, *OsARF12-L* for *OsARF12* and *OsARF12(WS)*, *OsARF16-U*, *OsARF16-L* for *OsARF16* and *OsARF16(WS)*, *OsARF17-U*, *OsARF17-L* for *OsARF17* and *OsARF17(WS)*, *OsARF25-U*, *OsARF25-L* for *OsARF25* and *OsARF25(WS)*. These coding sequences were cloned into *pGADT7* and transformed into yeast *AH109*. Yeast *Y187* containing *Osiaa23-3* or *Osiaa23-R5* were mated with *AH109* containing *OsARFs* or *OsARF(WS)s*, according to the manufacturer's protocol. Mated strains were spread on low stringency SD–Leu/–Trp and high stringency SD–Ade/–His/–Leu/–Trp. The sequences of primers are listed in Table S2. Interaction of *pGBKT7-53* and *pGADT7-T* was used as a positive control, and non-interaction of *pGBKT7-Lam* and *pGADT7-T* was used as a negative control.

### Self-activation test in the yeast system

The MATCHMAKER GAL4 Two-Hybrid System 3 (Clontech) was used to detect the autonomous activation of OsARF16 and OsARF16(WS). The coding sequences of *OsARF16* and *OsARF16(WS)* were amplified by primers *OsARF16-SU* and *OsARF16-SU*, and inserted into *pGBKT7* in-frame fused with the GAL4 DNA-BD. The fusion constructs were transformed into yeast strain *AH109* and selected on the minimal medium SD/-Trp and SD/-Trp-His-Ade to examine the reporter gene expression. The sequences of primers are listed in Table S2.

### PCR site-directed mutagenesis of *OsARFs*

PCR Site-Directed Mutagenesis of *OsARFs* were performed according to the instructions for the Fast Mutagenesis System (FM111, TransGenBiotech). The primers were *OsARF6-MU*, *OsARF6-ML* for *OsARF6*, *OsARF12-MU*, *OsARF12-ML* for *OsARF12*, *OsARF16-MU*, *OsARF1-ML* for *OsARF16*, *OsARF17-MU*, *OsARF17-ML* for *OsARF17* and *OsARF25-MU*, *OsARF25-ML* for *OsARF25*. The sequences of primers are listed in Table S2.

### Construction of vectors and transgenic plants development

The coding sequences of OsARF(WS)s were amplified by OsARF6-PU, OsARF6-PL for OsARF6(WS), OsARF12-PU, OsARF12-PL for OsARF12(WS), OsARF16-PU, OsARF16-PL for OsARF16(WS), OsARF17-PU, OsARF17-PL for OsARF17(WS) and OsARF25-PU, OsARF25-PL for OsARF25(WS). These coding sequences were cloned into a binary vector pHB, which had 35S promoter to drive these coding sequences. The sequences of primers are listed in Table S2.

These constructs were transformed into callus initiated from mature *Osiaa23-3* seeds by *Agrobacterium tumefaciens* (strain *EHA105*)-mediated transformation [21].

### RT-PCR analysis

For RT-PCR experiments, 5 µg of total RNA was denatured at 65°C for 5 min followed by quick chill on ice in a 14-μl reaction containing 1 µl oligo (dT)_12–18_(500 µg ml^−1^) primer, and 1 µl of 10 mM dNTP mixture (10 mM each dATP, dGTP, dCTP, and dTTP at neutral pH). After addition of 4 µl 5×reaction buffer (Promega), the reaction was incubated at 37°C for 2 min, 1 µl (200 units) of M-MLV RT^a^ (Promega) was added to the reaction and incubated at 42°C for another 50 min. After terminating, the reaction was heated at 70°C for 15 min for inactivating. The primers used were as follows: *OsARF6-RTU*, *OsARF6-RTL* for *OsARF6*, *OsARF12-RTU*, *OsARF12-RTL* for *OsARF12*, *OsARF16-RTU*, *OsARF16-RTL* for *OsARF16*, *OsARF17-RTU*, *OsARF17-RTL* for *OsARF17*, *OsARF25-RTU*, *OsARF25-RTL* for *OsARF25* and *OsACTIN-RTU*, *OsACTIN-RTL* for *OsACTIN*. The sequences of primers are listed in Table S2.

## Results

### Intragenic mutations rescued the defects of *Osiaa23-3* mutant

**Figure 1:**
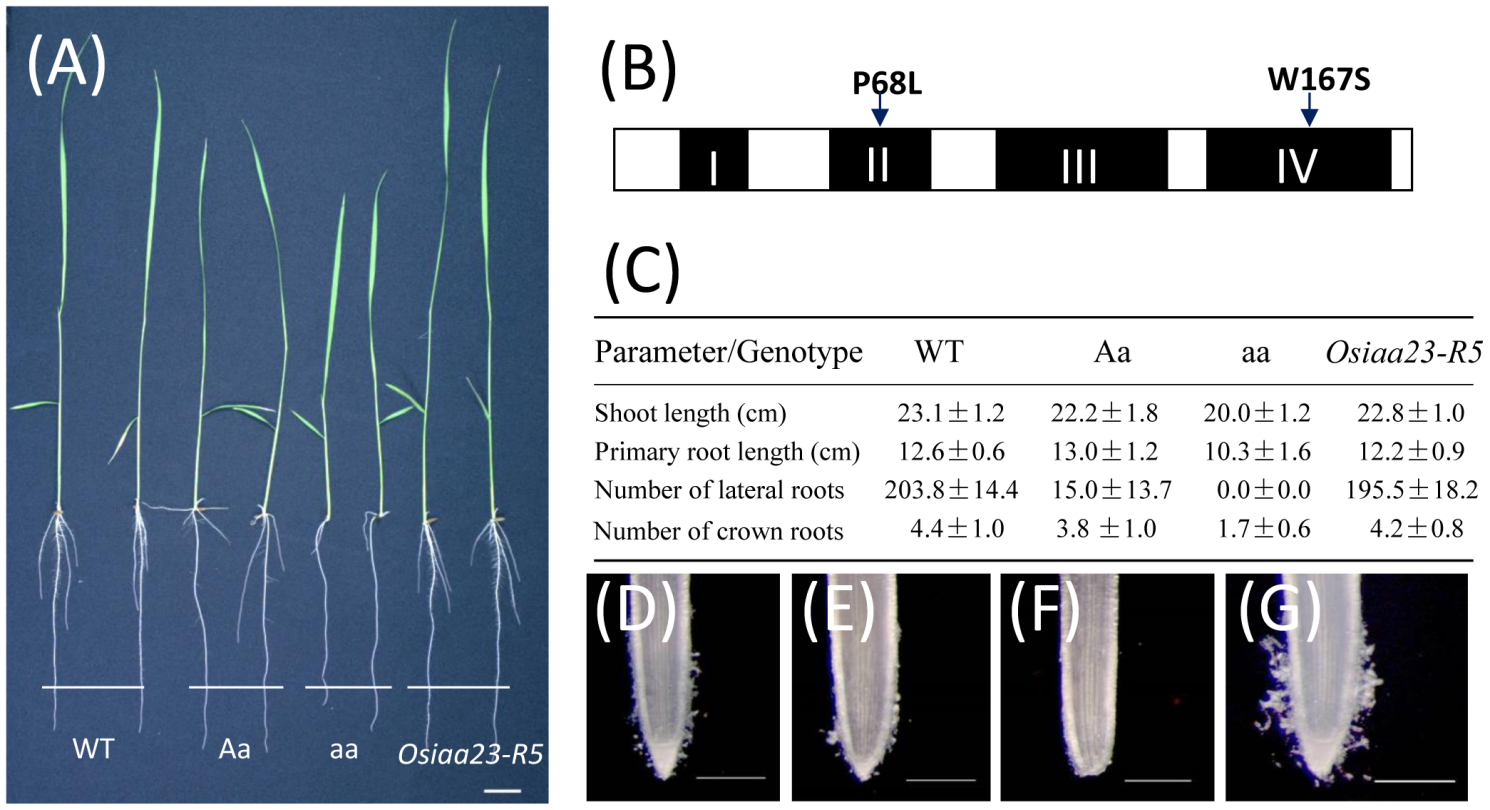
Intragenic mutation in Domain IV fully rescued the defects of *Osiaa23-3* mutant. (A) Phenotypes of 7-day-old seedlings of wild type (WT), heterozygous mutant of *Osiaa23-3* (Aa), homozygous mutant of *Osiaa23-3* (aa) and the suppressor of *Osiaa23-3*, *Osiaa23-R5*. Bar  = 2 cm. (B) Diagram of conserved domains of OsIAA23. Black rectangles marked by Roman numerals indicate four domains of OsIAA23, and two mutated sites in Domain II and Domain IV are marked by arrows. (C) Growth parameters of 7-day-old seedlings of WT, Aa, aa and *Osiaa23-R5*. (D-G) Root tips of 7-day-old seedlings of WT (D), Aa (E), aa (F) and *Osiaa23-R5* (G). Bars  = 250 µm.

In the previous research, we reported an auxin insensitive mutant designated as *Osiaa23*, which had multiple defects in both root and shoot development [4]. The *Osiaa23-3* is a weak allele of *Osiaa23*. The defects of *Osiaa23-3* are similar to that of *Osiaa23* reported before, except that *Osiaa23-3* can produce a few crown roots and can complete the life cycle (Figure 1). This weak allele was used in this research. In order to investigate different auxin signaling pathways corresponding to different defects, homozygous seeds of *Osiaa23-3* were mutagenized with ethyl methane sulfonate (EMS), and the M2 plants were screened for suppressors of *Osiaa23-3*. After the morphological screen of about 20,000 M2 plants, we successfully isolated six suppressors with different extent of recovery (Figure S1). Sequence analysis showed that all the suppressors had second site mutations in the *Osiaa23* gene. Furthermore, all the mutations changed the amino acids between Domain III and Domain IV of Osiaa23 (Figure S2), which is considered to be essential for the interaction between IAAs and ARFs [13].

### Amino acid substitution in Domain IV of Osiaa23 prevents the protein-protein interactions between Osiaa23 and OsARFs

Of all the suppressors isolated, *Osiaa23-R5* fully rescued all the defects in *Osiaa23-3* (Figure 1; Figure S3). This indicated that all the functions of OsARFs blocked by Osiaa23 have been released in *Osiaa23-R5*. Sequence analysis of *Osiaa23-R5* showed that a second point mutation occurred in *Osiaa23* resulting in an amino acid substitution from W to S in Domain IV (Figure 1B; Figure S2). To confirm that the amino acid substitution in Domain IV prevents the protein-protein interactions between Osiaa23 and OsARFs, we selected the appropriate OsARF candidates for yeast two-hybrid assay.

We chose OsARFs which had high expression in the root tip (Based on our previous microarray results of gene expression profile in rice root tip, Table S1) and the full-length cDNAs of *OsARF6, OsARF12, OsARF17 and OsARF25* were cloned. In addition, we also cloned the *OsARF16*, the homolog of *ARF7* and *ARF19*, which play key roles in lateral root initiation in *Arabidopsis* [22], [23]. To confirm the microarray results and explore their expression profiles in other tissues in rice, RT-PCRs were performed with total RNAs isolated from root, shoot base in young seedlings and stem, leaf, young panicle in adult plants. The analysis showed that all the five *OsARFs* are expressed in the selected tissues and none of the OsARFs have the tissue specific expression pattern (Figure S4).

**Figure 2:**
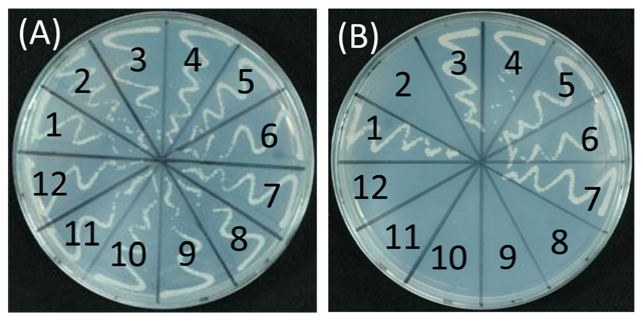
Amino acid substitution in Domain IV of Osiaa23 prevents the protein-protein interactions between Osiaa23 and OsARFs. Interactions between Osiaa23 and OsARFs, Osiaa23-R5 and OsARFs in the yeast two-hybrid system. 1, positive control; 2, negative control; 3, Osiaa23 + OsARF6; 4, Osiaa23 + OsARF12; 5, Osiss23 + OsARF16; 6, Osiaa23 + OsARF17; 7, Osiaa23 + OsARF25; 8, Osiaa23-R5 + OsARF6; 9, Osiaa23-R5 + OsARF12; 10, Osiaa23-R5 + OsARF16; 11, Osiaa23-R5 + OsARF17; 12, Osiaa23-R5 + OsARF25. Yeast was grown on medium without leucine and tryptophan (SD -Leu/ -Trp) as a contral (A) and medium without leucine, tryptophan, histidine and ade (SD -Trp/ -Leu/ -Ade/ -His) to test the protein-protein interactions (B).

The coding sequences of *Osiaa23*, *Osiaa23-R5* and five *OsARFs* (*OsARF6, OsARF12, OsARF16, OsARF17 and OsARF25*) were inserted into the yeast expression vectors *pGADT7* and *pGBKT7* respectively. All the transformed yeast cells formed colonies on the medium with histidine and ade (SD -Leu/ -Trp), indicating a successful transformation of these vectors (Figure 2A), while on the medium without histidine and ade (SD -Leu/ -Trp/ -His/ -Ade), only the transformed yeast cells expressing both *Osiaa23* and any of the *OsARFs* formed colonies, none of the transformed yeast cells formed colonies when *Osiaa23-R5* was used instead of *Osiaa23* (Figure 2B). These results indicated that Osiaa23 can interact with selected OsARFs and the W residue in Domain IV of Osiaa23 is crucial for the interactions.

### The W residue in Domain IV is conserved in both OsIAAs and OsARFs

**Figure 3:**
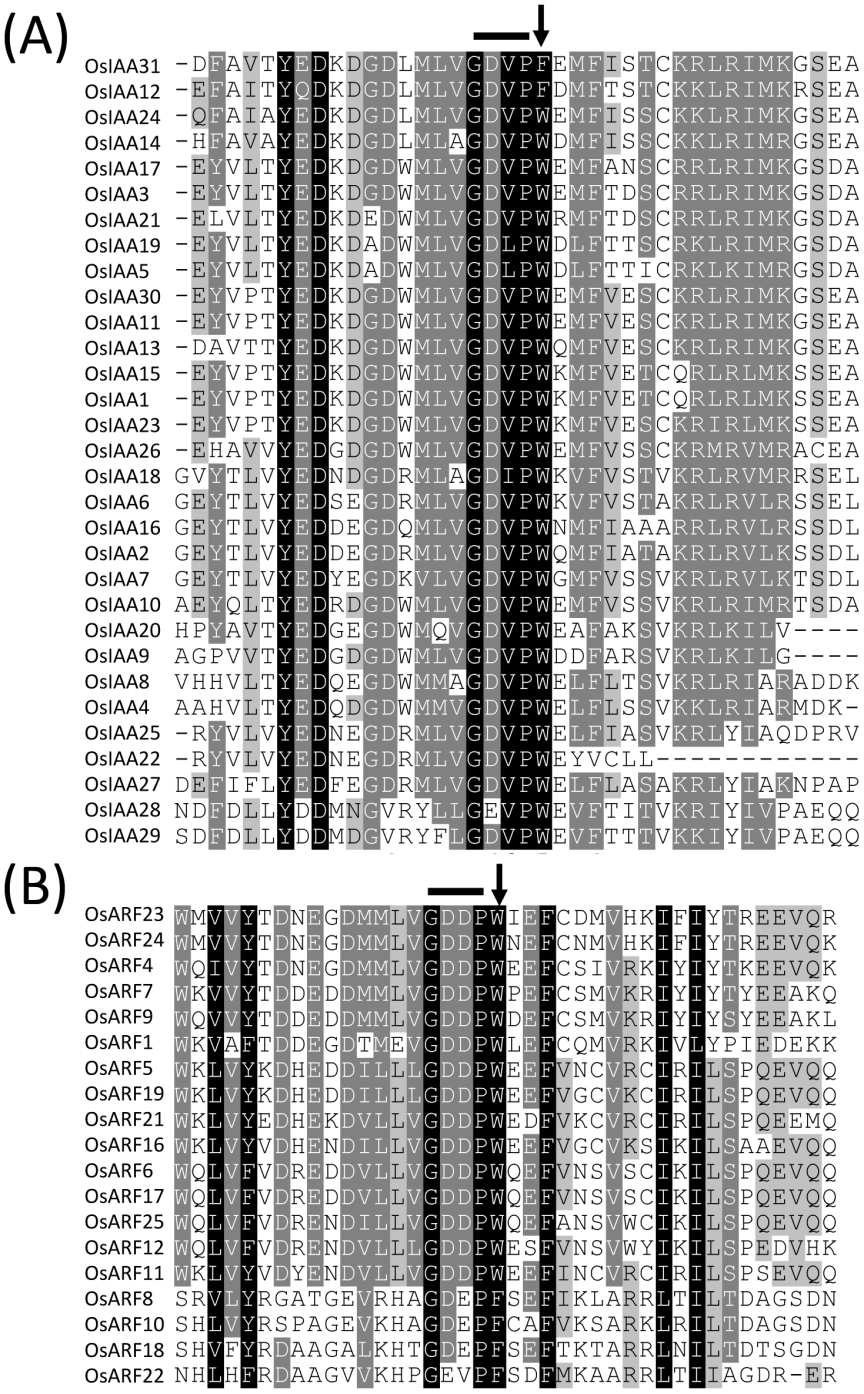
The W residue in Domain IV is conserved in both OsIAAs and OsARFs. (A) The alignment of Domain IV of OsAux/IAAs in rice, the conserved W is marked by the arrow. Conserved GDVP motif is indicated by thick line above the alignment. (B) The alignment of Domain IV of OsARFs in rice, the conserved W is marked by the arrow. Conserved GDDP motif is indicated by thick line above the alignment.

Because of the importance of W residue in Domain IV of OsIAA23, the alignment of Domain IV in all the 31 rice Aux/IAA proteins was performed using the ClustalX program. The result showed that the W residue was in the middle of Domain IV, and near the conserved motif GDVP [24]. Further analysis showed that the W residue is conserved among 29 OsIAAs. Although OsIAA12 and OsIAA31 have the Phenylalanine (F) residue instead of W, both F and W are aromatic amino acids and may have the similar properties (Figure 3A).

The protein-protein interactions between OsIAAs and OsARFs are mediated by the similar Domain III/IV in both protein families. So it is interesting to investigate whether OsARFs have the same conserved W residue in Domain IV. Of all the 25 OsARF proteins, 19 OsARFs have the conserved Domain IV [23]. The alignment of Domain IV in the 19 OsARFs was performed, and we found that the W residue also existed near the conserved motif GDDP in the Domain IV of OsARFs [24]. Although OsARF8, 10, 18 and 22 have F instead of W, they may have similar properties as mentioned above (Figure 3B).

### The W residue in Domain IV of OsARFs is crucial for the protein-protein interactions between Osiaa23 and OsARFs

**Figure 4:**
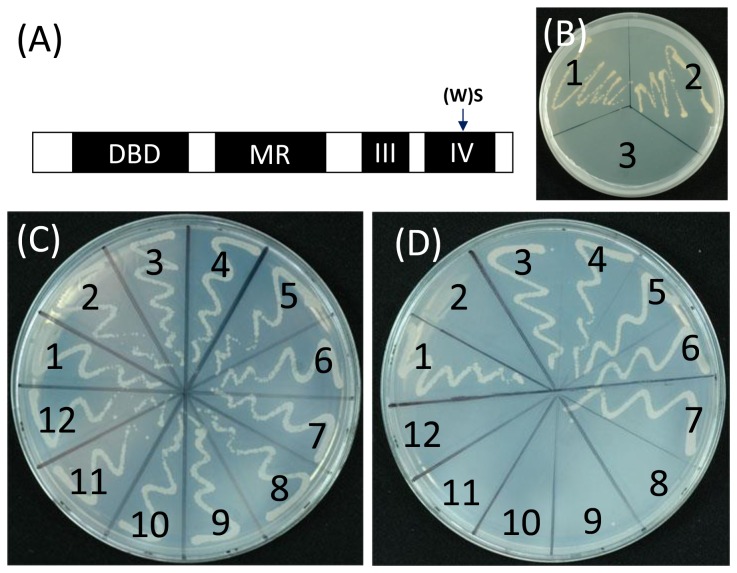
The W residue in Domain IV of OsARFs is crucial for the protein-protein interactions between Osiaa23 and OsARFs. (A) Diagram of conserved domains of OsARF protein. Black rectangles indicate four domains of OsARF, DBD and MR represent DNA binding Domain and middle region respectively, III and IV represent Domain III and Domain IV, which are similar to that of OsIAA. The substitution from W to S in Domain IV is indicated by arrow. (B) Self-activation test of OsARF16 and OsARF16(WS), which has an amino acid substitution from W to S in Domain IV. The transformed yeast was grown on medium without tryptophan, histidine and ade (SD -Trp/ -His/ -Ade). 1, OsARF16; 2, OsARF16(WS); 3, negative control. (C-D) Interactions between Osiaa23 and OsARFs, Osiaa23 and OsARF(WS)s in the yeast two-hybrid system. 1, positive control; 2, negative control; 3, Osiaa23 + OsARF6; 4, Osiaa23 + OsARF12; 5, Osiss23 + OsARF16; 6, Osiaa23 + OsARF17; 7, Osiaa23 + OsARF25; 8, Osiaa23 + OsARF6(WS); 9, Osiaa23 + OsARF12(WS); 10, Osiaa23 + OsARF16(WS); 11, Osiaa23 + OsARF17(WS); 12, Osiaa23 + OsARF25(WS). Yeast was grown on medium without leucine and tryptophan (SD –Leu/ -Trp) as a contral (C) and medium without leucine, tryptophan, histidine and ade (SD -Trp/ -Leu/ -Ade/ -His) to test the protein-protein interactions (D).

To examine whether the conserved W residue in Domain IV of OsARFs affects the protein-protein interactions between Osiaa23 and OsARFs, the W residue in the Domain IV of OsARF6, 12, 16, 17 and 25 was exchanged with S respectively, as the same change occurred in Osiaa23-R5 (Figure 4A). The coding sequences of mutated *OsARF6(WS)*, *OsARF12(WS)*, *OsARF16(WS)*, *OsARF17(WS)* and *OsARF25(WS)* were inserted into yeast expression vector *pGBKT7* and transformed into yeast cells along with *Osiaa23*. The results showed that none of the transformed yeast cells expressing *OsARF6(WS)-Osiaa23*, *OsARF12(WS)-Osiaa23*, *OsARF16(WS)-Osiaa23*, *OsARF17(WS)-Osiaa23*, *OsARF25(WS)-Osiaa23* formed colonies (Figure 4C, D). These results showed that the W residue in Domain IV of OsARFs is crucial for the protein-protein interactions between Osiaa23 and OsARFs.

### Mutated OsARF(WS)s rescued different defects of *Osiaa23-3*

To examine the transcriptional activities of the OsARF(WS)s mutated in Domain IV, the autonomous gene activation test was performed in the yeast system. The full length cDNA of *OsARF16* and *OsARF16(WS)* were fused to the DNA-BD of the yeast transcription factor GAL4 and transformed into yeast strain *AH109*. Both strains with *OsARF16* and *OsARF16(WS)* could grow well on SD -Trp/ -His/ -Ade (Figure 4B). This result indicates that the mutation in Domain IV does not affect the transcriptional activities of OsARFs.

In order to investigate the functions of OsARFs suppressed in *Osiaa23*, the coding sequences of mutated *OsARF(WS)s* were driven by the constitutive promoter (*35S*) and transformed into *Osiaa23-3*. All the transgenic lines were confirmed by RT-PCR to insure their enhanced expressions (Figure S5). The phenotypes of transgenic rice were compared with that of *Osiaa23-3*.

**Figure 5:**
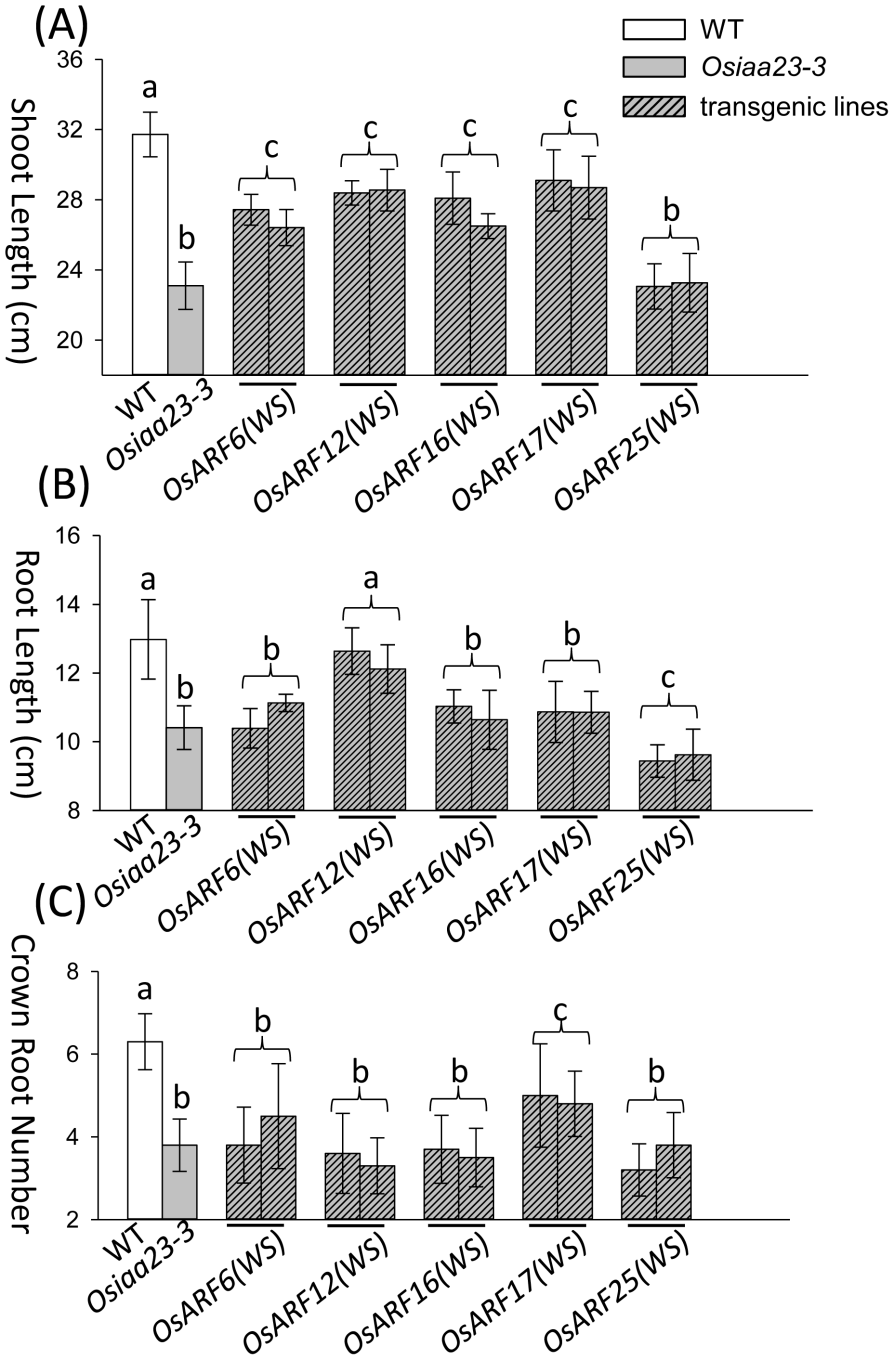
Mutated OsARF(WS)s rescued different defects of *Osiaa23-3*. The phenotypes of transgenic rice, over expressing *OsARF(WS)s* in the *Osiaa23-3* background. Two independent lines of transgenic rice over expressing *OsARF(WS)s* are compared with WT and *Osiaa23-3* in the aspects of shoot length (A), root length (B) and crown root number (C). Statistically distinct groups are marked by a, b and c (n = 10).

None of the transgenic rice rescued the root cap or lateral root defects of *Osiaa23-3* (Figure S5; Figure S6). Further analysis revealed that over expression of *OsARF6(WS)*, *OsARF12(WS)*, *OsARF16(WS)*, and *OsARF17(WS)* in *Osiaa23-3* partially rescued the shoot length of the mutant, while over expression of *OsARF25(WS)* had no effect to the shoot length of *Osiaa23-3* (Figure 5A; Figure S5). In the aspect of root length, over expression of *OsARF12(WS)* in *Osiaa23-3* fully rescued the root length, while over expression of *OsARF25(WS)* reduced root growth in *Osiaa23-3* (Figure 5B; Figure S5). Over expression of *OsARF17(WS)* partially rescued the number of crown roots as compared with *Osiaa23-3* (Figure 5C).

## Discussion

### Intragenic suppressor *Osiaa23-R5* fully rescued all the defects of *Osiaa23-3*

These is no information on the 3D structures of Domain III/IV in Aux/IAA or ARF proteins, and little is known about which amino acid residues are important for protein-protein interactions. Current knowledge comes from intragenic suppressors of gain-of-function *iaa* mutants that specific amino acid substitutions in Domain III/IV revert the mutant phenotypes to wild type phenotypes, presumably by suppressing protein-protein interactions [24]. Although intragenic suppressors of *iaa* mutants have been reported in *Arabidopsis*, none of them fully rescued the defects of *iaa* mutants, this indicated that these amino acid residues in Domain III/IV may not vital for protein-protein interactions [25], [26]. In this study, we described an intragenic suppressor of *Osiaa23* mutant, *Osiaa23-R5*, which fully rescued all the defects of *Osiaa23-3*. Sequence analysis revealed a second site mutation in *Osiaa23-R5*, resulting in an amino acid substitution in Domain IV. Yeast two-hybrid experiments showed that Osiaa23 can interact with selected OsARFs, while the Osiaa23-R5, which has an amino acid substitution in Domain IV, cannot interact with any of these OsARFs. These results partially explained the reasons why the amino acid substitution of W in Domain IV of Osiaa23-R5 fully rescued the defects of *Osiaa23-3*, and indicated that W residue in Domain IV of OsIAA23 may crucial for protein-protein interactions.

It was originally proposed that the Domain III/IV of Aux/IAA and ARF families contain a secondary structure consisting of a beta sheet (β) followed by two alpha helices (α). It was suggested that the predicted amphipathic βαα motif might function in dimerization [24]. Interestingly, the W residue is at the beginning of α2 motif. This implied that α2 motif may play an important role in protein-protein interactions between Aux/IAA and ARF families. Studies of other suppressors showed that although βαα motif has an important role in dimerization, residues outside of βαα motif may also involve in protein-protein interactions. One suppressor, *Osiaa23-R3*, has an amino acid substitution between Domain III and Domain IV, which is outside of βαα motif, shows weak extent of recovery (Figure S1).

### The verified functions of ARFs

Most of knowledge about the functions of ARFs has been revealed by forward genetic approaches. Examination of phenotypic defects in knock-out *arf* mutants is a direct way to find out the functions of ARFs.

*arf2/hss* has defects in apical hook formation and has increased seed size [27], [28], *arf3/ett* lost the abaxial identity in the gynoecium [29]; *arf5/mp* has defects in embryo development and vascular tissue formation [30], *arf7/nph4* has defects in hypocotyl tropisms and resistance to auxin and ethylene [31]; *arf8* uncouples fruit development from fertilization [32], *arf19* shows insensitivity to auxin and ethylene [33]. Identification and characterization of T-DNA insertion lines for 18 of the *ARFs* showed that most of the lines fail to show an obvious growth phenotype except for the previously identified *arf2/hss*, *arf3/ett*, *arf5/mp*, and *arf7/nph4* mutants, suggesting that there are functional redundancies among the ARF proteins [34].

Double mutants of closely related ARFs generally show much stronger phenotypes than single mutants. *arf1 arf2* double mutant enhance the defects of *arf2* [27], [35]. *arf3 arf4* double mutant has reduced abaxial identity in all organs compared with defects only in the gynoecium [36]. *arf5 arf7* double mutant enhances the defects in the embryo patterning and vasculature of *arf5* [37]. *arf6 arf8* double mutant shows flower arrested development and is completely infertile, while both *arf6* and *arf8* single mutants only show delayed flower maturation and reduced fertility [38]. *arf7 arf19* double mutant shows a strong auxin-related phenotype and severely impaired lateral root formation and abnormal gravitropism in both hypocotyl and root. These defects were not observed in the *arf7* and *arf19* single mutants [34], [39]. *arf10* and *arf16* single mutants have no defects, while double mutant of *arf10 arf16* shows root cap defects and abnormal root gravitropism [40].

A family of *OsARFs* has been described in rice with 25 *OsARFs* compared with 23 *ARFs* in *Arabidopsis* [23], [41]. The phylogenetic relationship analysis showed that the organization of rice *OsARFs* were very similar to that of *Arabidopsis ARFs*, implying that rice and *Arabidopsis ARFs* were derived from a common ancestor, and they existed before the divergence of monocots and dicots [23].

Limited information has been obtained in the functions of *OsARFs* in rice. *OsARF1* (or *OsARF23*) is the first *OsARF* gene described in rice, and it is closely related to *ARF1* and *ARF2* in *Arabidopsis*. Knock-down of *OsARF1* has defects in vegetative and reproductive development, which is similar to the double mutant of *arf1 arf2* in *Arabidopsis* [35], [42], [43]. OsARF12 has been proved to regulate root elongation and affect iron accumulation in rice [44]. On the other hand, *OsARF11* was the orthologue of *ARF5*, and *OsARF16* was the orthologue of *ARF7* and *ARF19*. However, transposon insertions in *OsARF11* and *OsARF16* do not show similar defects as *arf5* and *arf7 arf19* double mutant in *Arabidopsis* [23]. Interestingly, OsARF16 is required for both auxin and phosphate starvation response in rice [45]. These results showed similarities and differences of ARF functions between rice and *Arabidopsis*.

Recent studies showed that four *OsARFs* (*OsARF6*, *OsARF12*, *OsARF17* and *OsARF25*) are negatively controlled by miR167. The transgenic rice over expressing *miR167* showed a substantial decrease in the expression of these four *OsARF* genes. Moreover, the transgenic rice were small in stature with remarkably reduced tiller number [46]. These results showed that these four OsARFs are important to the normal growth and development, while the functions of different OsARFs are still to be characterized.

### An indirect way to investigate functions of OsARFs

*Osiaa23* exhibits pleiotropic defects in both shoot and root development, this means that the stabilized Osiaa23 restricted many OsARFs, which was supported by our yeast two-hybrid experiments (Figure 2B, C). In this research, the mutated OsARF(WS)s can be released from the inhibition of Osiaa23 and maintain the transcriptional activities. These results provide an indirect way to investigate functions of OsARFs.

The mutated *OsARF(WS)s* were transformed into *Osiaa23-3* mutant, and the phenotypes of transgenic rice were compared with that of *Osiaa23-3*. A large-scale analysis of the Aux/IAA-ARF interactome predicted a strong buffering capacity of the Aux/IAA-ARF network in the shoot apex of *Arabidopsis* [47]. In our research, over expression of *OsARF6(WS)*, *OsARF12(WS)*, *OsARF16(WS)* and *OsARF17(WS)* partially rescued the shoot length of *Osiaa23-3*, this implies that these OsARFs may be redundantly involved in the shoot development. Over expression of *OsARF12(WS)* rescued the root length of *Osiaa23-3*, this implies that OsARF12 may be involved in the root development. This is in agreement with the recent finding that OsARF12 regulates root elongation in rice [44]. Over expression of *OsARF17(WS)* partially rescued the crown root number of *Osiaa23-3*, this implies that OsARF17 may be involved in the crown root initiation in rice. Interestingly, over expression of *OsARF25(WS)* didn’t rescue any defects in *Osiaa23-3*, hence, the root length was even shorter. This implies that OsARF25 may function as a negative regulator in rice root development.

Over expression of site-specific mutated *OsARF(WS)s* rescued several defects of *Osiaa23-3*, while none of the transgenic rice rescued the root cap or lateral root defects. Considering that auxin gradient is needed in both the initiation of lateral root and maintenance of root apical meristem [48], [49], native promoter of *OsARFs* may be required to rescue the root development of *Osiaa23-3*. Alternatively, considering the possibility of false positives in yeast two-hybrid experiments [24], the selected five OsARFs may not interact with Osiaa23 *in vivo*, and the rest of OsARFs may be involved in these process or more than one OsARFs should work together to regulate these developments.

## Supporting Information

Figure S1 **Suppressors of** ***Osiaa23-3*** **with different extent of recovery.** (A) Root phenotypes of 7-day-old suppressors of *Osiaa23-3*. 1, wild type; 2, *Osiaa23-3*; 3, *Osiaa23-R5*, which fully rescued all the defects of *Osiaa23-3*; 4-8, the rest of the suppressors, which partially rescued defects of *Osiaa23-3*. 4, *Osiaa23-R1*; 5, *Osiaa23-R2*; 6, *Osiaa23-R3*; 7, *Osiaa23-R4*; 8, *Osiaa23-R6*. Bar  = 2 cm. (B) Lateral root numbers of revertant mutants of *Osiaa23*. 1, wild type; 2, *Osiaa23*, which has no lateral root; 3, *Osiaa23-R5*; 4-8, the rest of the suppressors. 4, *Osiaa23-R1*; 5, *Osiaa23-R2*; 6, *Osiaa23-R3*; 7, *Osiaa23-R4*; 8, *Osiaa23-R6*.
(TIF)

Figure S2 **The mutation sites of intragenic suppressors of** ***Osiaa23-3*** **.** The amino acid sequence of OsIAA23, four domains of OsIAA23 are underlined. Red arrow in Domain II represents the mutation site of *Osiaa23-3*, the other 6 arrows represent mutation sites of six intragenic suppressors, these sites are distributed between Domain III and Domain IV. The substitutions of K to M, V to E, A to G, M to T, W to S and R to Q result in the phenotypes of *Osiaa23-1*, *Osiaa23-2*, *Osiaa23-3*, *Osiaa23-4*, *Osiaa23-5* and *Osiaa23-6* respectively.
(TIF)

Figure S3 **The magnification of** **Figure 1(A)** **.**
(TIF)

Figure S4 **The expression patterns of selected** ***OsARFs*** **.** Semi-quantitative RT-PCR analysis of *OsARF6*, *OsARF12*, *OsARF16*, *OsARF17* and *OsARF25* expressions in root (R), stem-base (SB) of 7-d-old wild-type seedlings, and in stem (S), leaf (L) and panicle (P) of adult plants.
(TIF)

Figure S5 **Phenotypes of transgenic rice.** Phenotypes of transgenic rice over expressing *OsARF6(WS)* (A), *OsARF12(WS)* (B), *OsARF16(WS)* (C), *OsARF17(WS)* (D) and *OsARF25(WS)* (E) in the *Osiaa23-3* background. From left to right are wild type, *Osiaa23-3* and two independent transgenic lines in the *Osiaa23-3* background. Bars  = 2 cm. The lowers are RT-PCR results of these transgenic lines.
(TIF)

Figure S6 **Root tips of transgenic rice over expressing** ***OsARF(WS)s*** **.** From left to right are wild type, *Osiaa23-3* mutant and five transgenic rice over expressing different *OsARF(WS)s* in the background of *Osiaa23-3*. None of the transgenic rice recovered the root tip defect. Bars  = 0.5 mm.
(TIF)

Table S1 **The microarray results of gene expression profile in rice root tip.** Total RNA was extracted from root tips (1 cm) of 7-day-old rice. The experiment included two biological replicates (Signal 1 and Signal 2). Microarray analysis was carried out using an Affymetrix technology platform and Affymetrix GeneChip rice genome array.
(ZIP)

Table S2 **The sequences of primers used in this paper.**
(XLSX)
