# Supplementary figures and images for: Intragenic Suppressor of Osiaa23 Revealed a Conserved Tryptophan Residue Crucial for Protein-Protein Interactions
Source: PLoS One. 2014 Jan 15;9(1):e85358. doi: 10.1371/journal.pone.0085358 (PMC3893212; doi:10.1371/journal.pone.0085358)

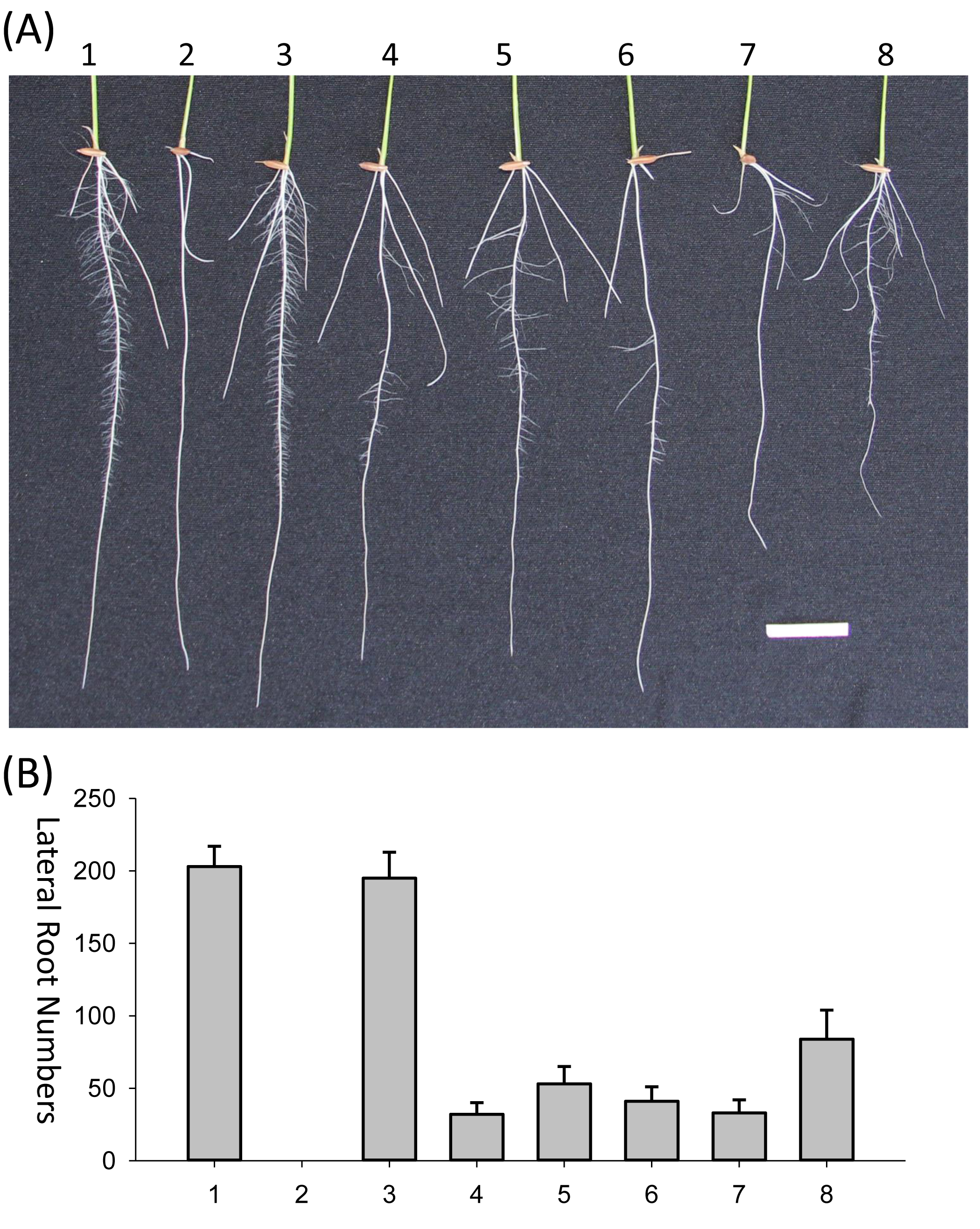

Supplement: Figure S1 — Suppressors of Osiaa23-3 with different extent of recovery. (A) Root phenotypes of 7-day-old suppressors of Osiaa23-3. 1, wild type; 2, Osiaa23-3; 3, Osiaa23-R5, which fully rescued all the defects of Osiaa23-3; 4-8, the rest of the suppressors, which partially rescued defects of Osiaa23-3. 4, Osiaa23-R1; 5, Osiaa23-R2; 6, Osiaa23-R3; 7, Osiaa23-R4; 8, Osiaa23-R6. Bar = 2 cm. (B) Lateral root numbers of revertant mutants of Osiaa23. 1, wild type; 2, Osiaa23, which has no lateral root; 3, Osiaa23-R5; 4-8, the rest of the suppressors. 4, Osiaa23-R1; 5, Osiaa23-R2; 6, Osiaa23-R3; 7, Osiaa23-R4; 8, Osiaa23-R6. (TIF) [file pone.0085358.s001.tif]

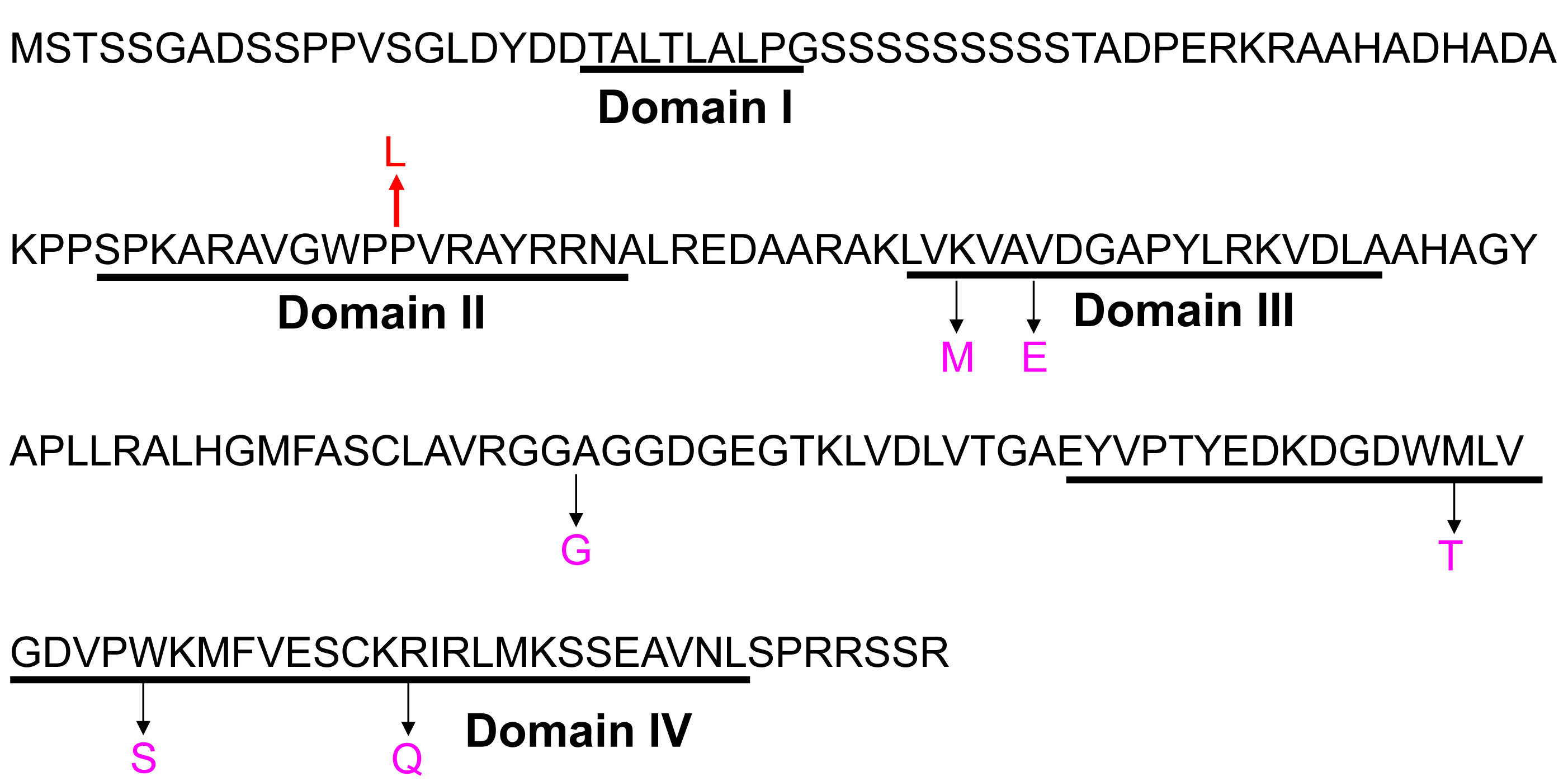

Supplement: Figure S2 — The mutation sites of intragenic suppressors of Osiaa23-3 . The amino acid sequence of OsIAA23, four domains of OsIAA23 are underlined. Red arrow in Domain II represents the mutation site of Osiaa23-3, the other 6 arrows represent mutation sites of six intragenic suppressors, these sites are distributed between Domain III and Domain IV. The substitutions of K to M, V to E, A to G, M to T, W to S and R to Q result in the phenotypes of Osiaa23-1, Osiaa23-2, Osiaa23-3, Osiaa23-4, Osiaa23-5 and Osiaa23-6 respectively. (TIF) [file pone.0085358.s002.tif]

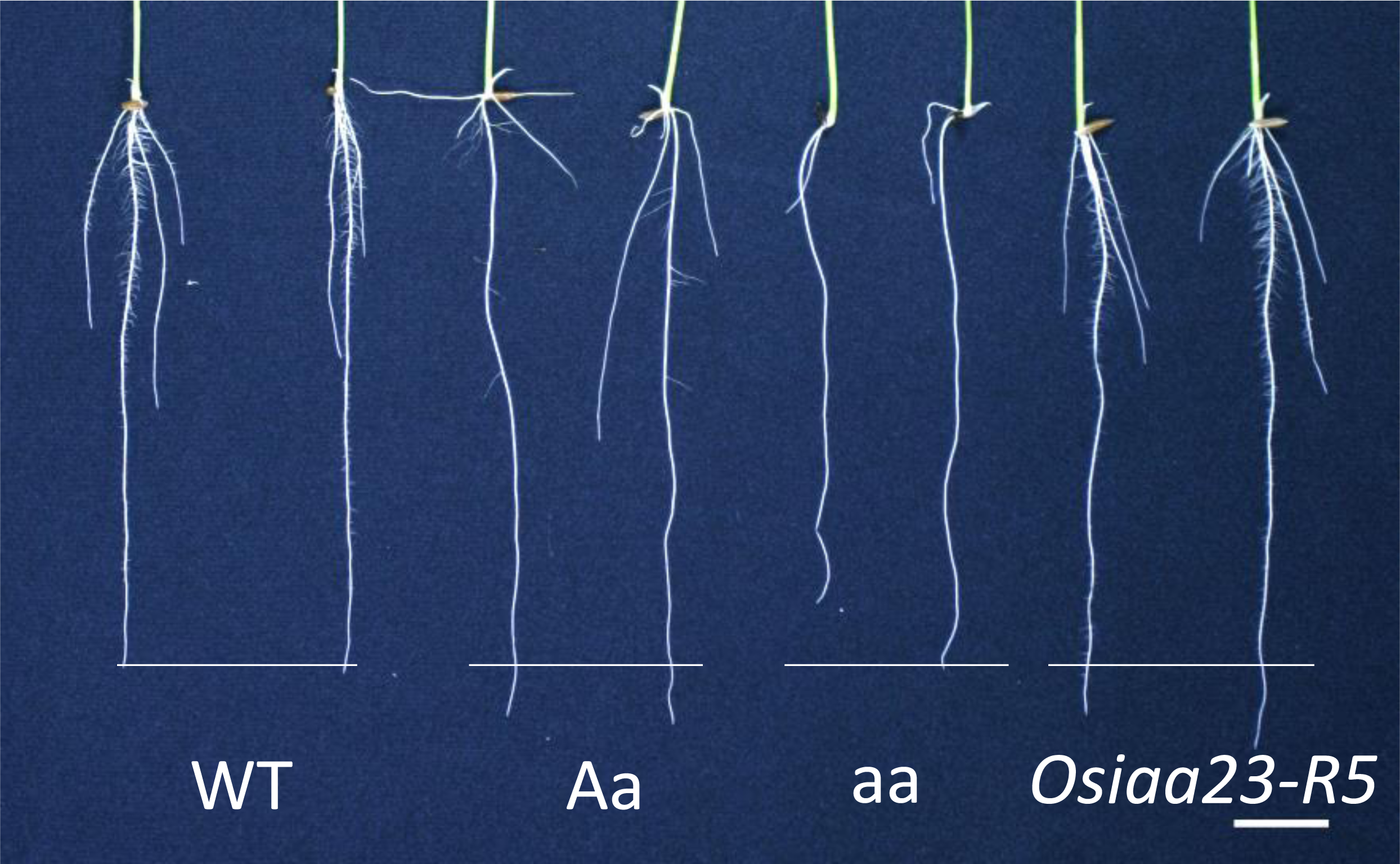

Supplement: Figure S3 — The magnification of Figure 1(A) . (TIF) [file pone.0085358.s003.tif]

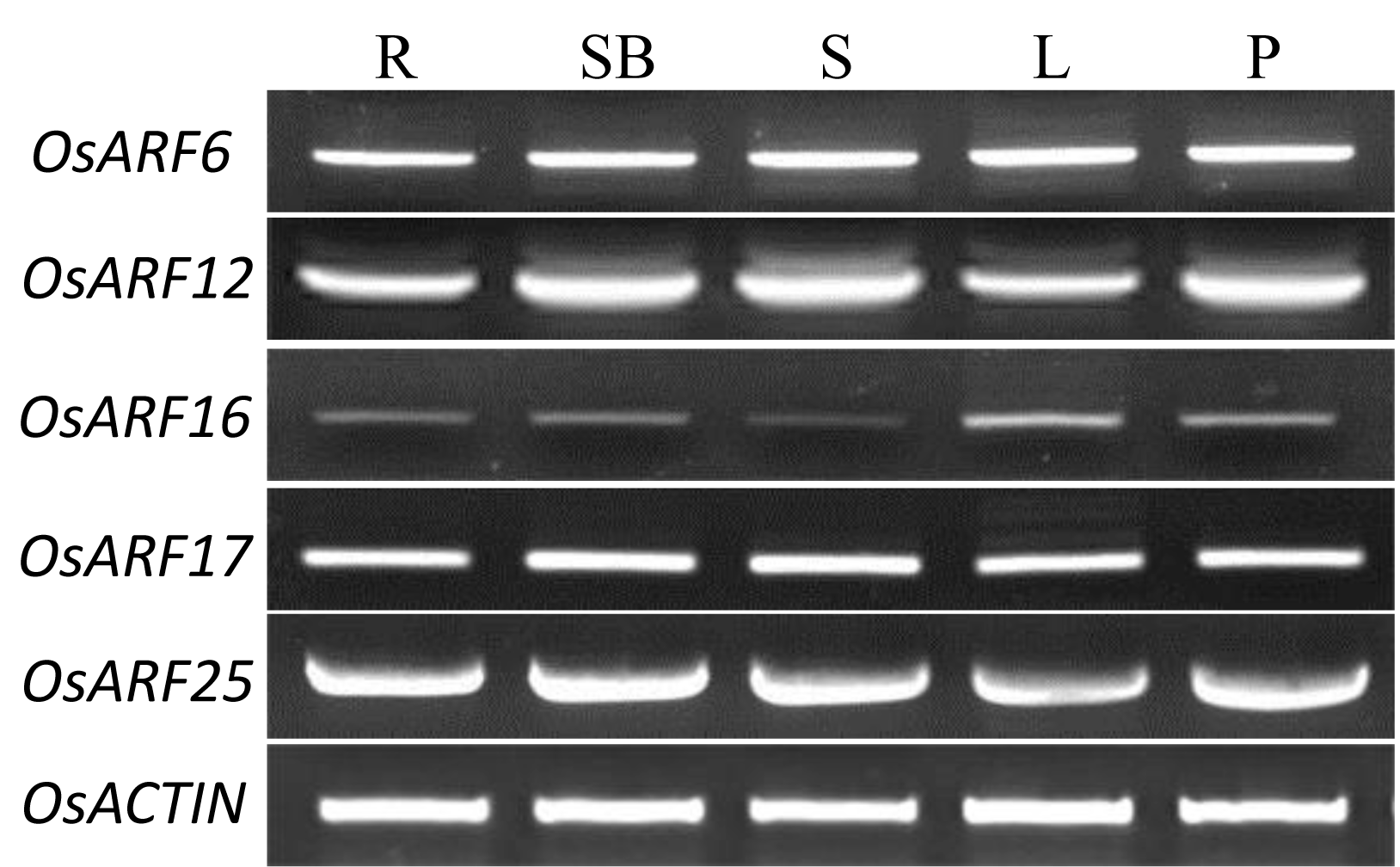

Supplement: Figure S4 — The expression patterns of selected OsARFs . Semi-quantitative RT-PCR analysis of OsARF6, OsARF12, OsARF16, OsARF17 and OsARF25 expressions in root (R), stem-base (SB) of 7-d-old wild-type seedlings, and in stem (S), leaf (L) and panicle (P) of adult plants. (TIF) [file pone.0085358.s004.tif]

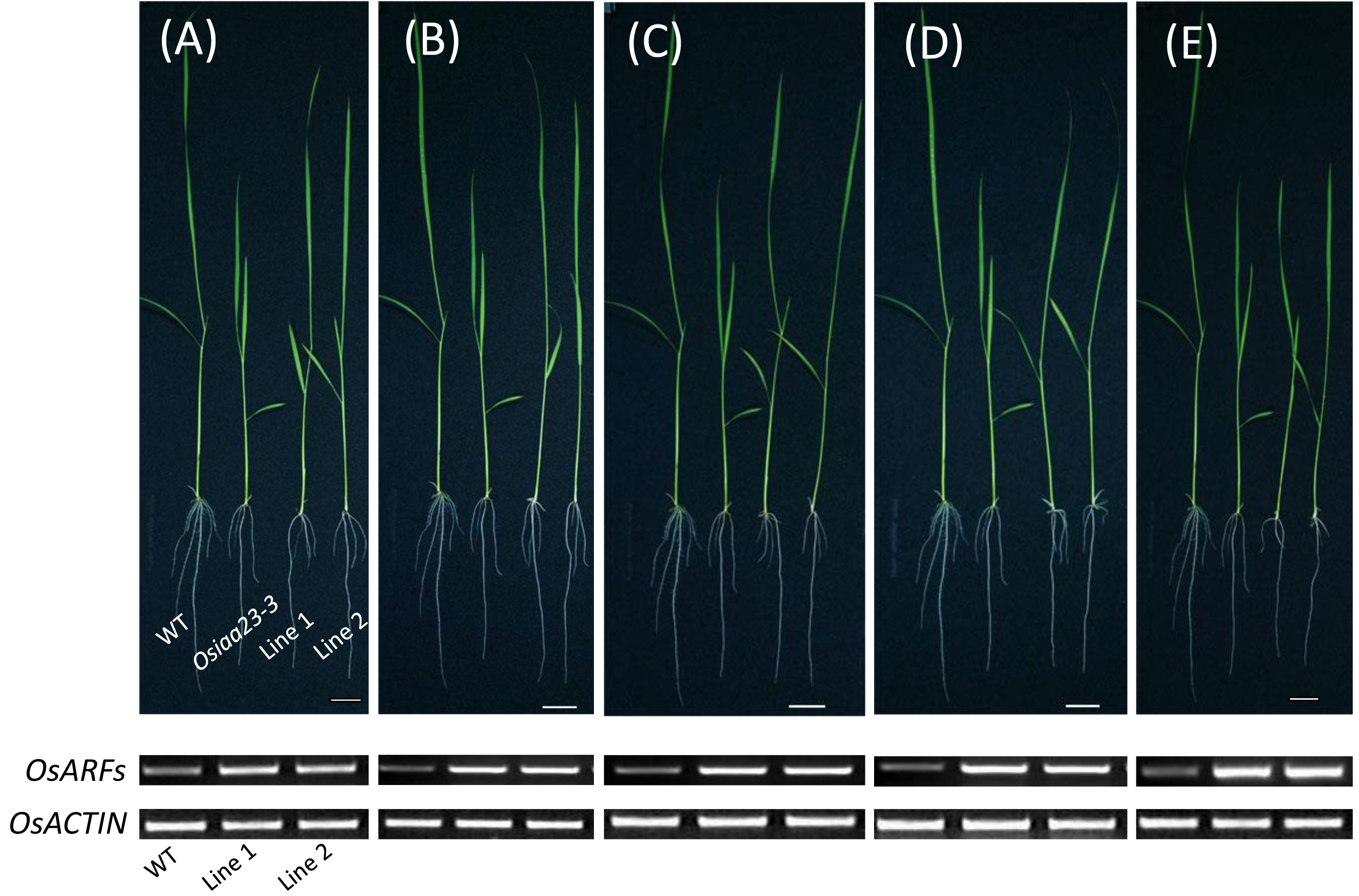

Supplement: Figure S5 — Phenotypes of transgenic rice. Phenotypes of transgenic rice over expressing OsARF6(WS) (A), OsARF12(WS) (B), OsARF16(WS) (C), OsARF17(WS) (D) and OsARF25(WS) (E) in the Osiaa23-3 background. From left to right are wild type, Osiaa23-3 and two independent transgenic lines in the Osiaa23-3 background. Bars = 2 cm. The lowers are RT-PCR results of these transgenic lines. (TIF) [file pone.0085358.s005.tif]

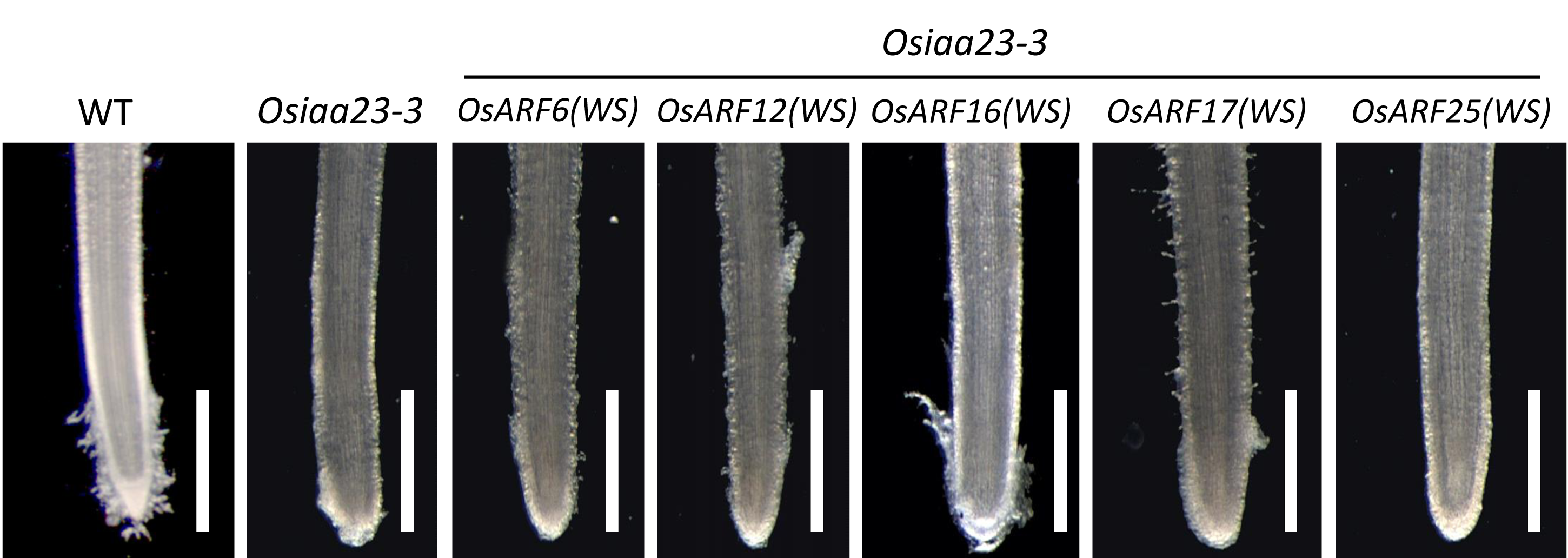

Supplement: Figure S6 — Root tips of transgenic rice over expressing OsARF(WS)s . From left to right are wild type, Osiaa23-3 mutant and five transgenic rice over expressing different OsARF(WS)s in the background of Osiaa23-3. None of the transgenic rice recovered the root tip defect. Bars = 0.5 mm. (TIF) [file pone.0085358.s006.tif]
